# Exploiting the roles of nitrogen sources for HEA increment in *Cordyceps cicadae*

**DOI:** 10.3389/fmicb.2024.1384027

**Published:** 2024-05-13

**Authors:** Kexin Zhu, Haihua Ruan, Tao Wu, Hongyang Zhang, Wenying Han, Qiqing Shen

**Affiliations:** Tianjin Key Laboratory of Food Science and Biotechnology, College of Biotechnology and Food Science, Tianjin University of Commerce, Tianjin, China

**Keywords:** *Cordyceps cicadae*, source of nitrogen, nucleosides, N^6^-(2-hydroxyethyl)-adenosine, transcriptome analysis

## Abstract

*Cordyceps cicadae*, as a new food ingredient, is a valuable edible and medicinal fungi. However, its resources are severely depleted due to environmental limitations and excessive harvesting practices. N^6^-(2-hydroxyethyl) adenosine (HEA), as an important product of *Cordyceps cicadae*, has the potential to be used in medical industry due to its diverse disease curing potential. However, the disclosure of HEA synthesis still severely limited its application until now. In this study, the kinetic curves for adenosine and HEA under shaker fermentation were explored. The kinetics of HEA and adenosine production exhibited a competitive pattern, implicating a possibility of sharing a same step during their synthesis. Due to HEA as a derivative of nitrogen metabolism, the effect of different nitrogen sources (peptone, yeast extract, ammonium sulfate, diammonium oxalate monohydrate, ammonium citrate dibasic, and ammonium citrate tribasic) on HEA production in *Cordyceps cicadae* strain AH 10-4 had been explored under different incubation conditions (shaker fermentation, stationary fermentation, and submerged fermentation). Our results indicated that the complex organic nitrogen sources were found to improve the accumulation of HEA content under shaker fermentation. In contrast, the optimal nitrogen source for the accumulation of HEA under stationary fermentation and submerged fermentation was ammonium citrate tribasic. But submerged fermentation obviously shortened the incubation time and had a comparable capacity of HEA accumulation by 2.578 mg/g compared with stationary fermentation of 2.535 mg/g, implicating a possibility of scaled-up production of HEA in industry by submerged fermentation. Based on the dramatic HEA production by ammonium sulfate as nitrogen resources between stationary and shaker fermentations, alanine, aspartate and glutamate as well as arginine metabolic pathway were related to the production of HEA by comparative transcriptome. Further investigation indicated that glutamic acid, which is an analog of Asp, showed an optimum production of HEA in comparison with other amino acids.

## Graphical abstract

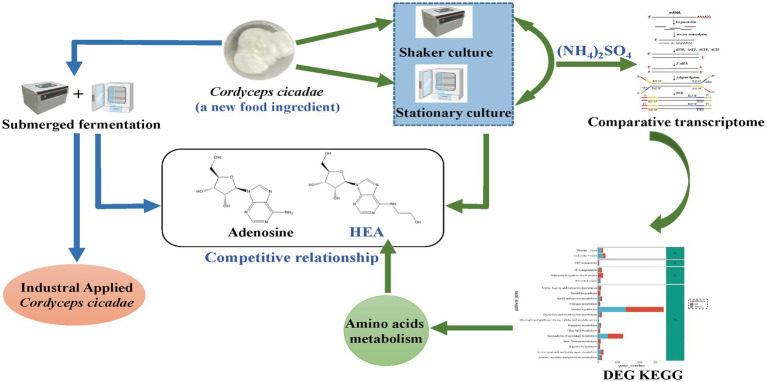

## 1 Introduction

*Cordyceps cicadae* (*C. cicadae*), a high-quality *cordyceps* similar to *Cordyceps sinensis* and *Cordyceps militaris* (Cheng et al., 2012), is a fungi abundant with bioactive compounds such as nucleoside, ergosterol, cordycepic acid, and polysaccharide, etc. It is reported possessing remarkable clinical activities including anti-tumor (Yang and Zhang, 2016), immune regulation (Zheng et al., 2022), and significant resistance to renal failure (Zhu et al., 2011) etc. *Cordyceps cicadae* has been used as an important herbal medicine for more than 1,600 years (Nxumalo et al., 2020). Particularly since 2021, artificially cultivated *C. cicadae* is approved as a new food ingredient (National Health Commission, 2020), expanding the application field of *C. cicadae* from traditional Chinese medicine to food, which indicates a promising future for its wide-ranging applications.

The majority of *Cordyceps cicadae* is obtained from the wild, particularly in Southeast Asia (Xie et al., 2023). Wild Cicada flowers require specific environments and parasitic hosts to grow, resulting in its rare resources (Zeng et al., 2021). Given its use as both medicine and food, the increasing demand for this valuable fungus has led to unsustainable harvesting practices (Liu et al., 2008). Therefore, mycelial fermentation to produce the bioactive compounds need to be investigated as an alternative substitute for wild *C. cicadae. Paecilomyces cicadae* (*P. cicadae*), reported as the anamorph stage of *C. cicadae*, is mainly used as the strain for artificial culture of *C. cicadae*, and this kind of cultivated *P. cicadae* has been shown controllable quality (Li et al., 2019). The artificial cultivation techniques of *C. cicadae* comprise liquid fermentation (Horng et al., 2021), solid fermentation (Lu et al., 2017; Shi et al., 2022), and artificial culture of alternative host insects (Liu et al., 2018).

**Figure 1:**
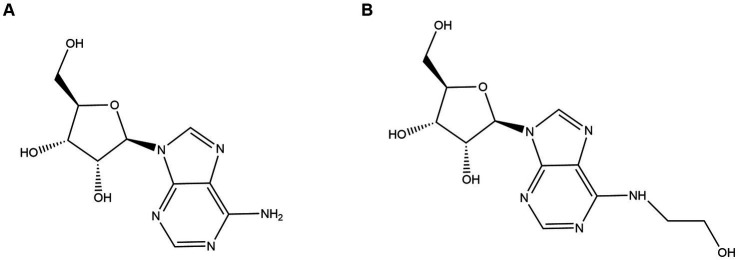
Structure diagrams of **(A)** adenosine and **(B)** N^6^-(2-hydroxyethyl) adenosine.

Adenosine (Figure 1A) and N^6^-(2-hydroxyethyl) adenosine (HEA) (Figure 1B), as purine nucleosides, are the major bioactive constituents of *Cordyceps*. As far as 1983, HEA was first isolated and purified from the fermentation broth of cultured *Cordyceps* (Furuya et al., 1983). Since then, adenosine and HEA have become important indicators for assessing the quality of *Cordyceps* products. A wild cicada flowers from Anji, Zhejiang city in China was isolated and purified in our laboratory (Huang et al., 2021), which was identified as a new strain of *C. cicadae* by phylogenetic tree with higher yield of adenosine and HEA as compared to previous studies (Chunyu et al., 2019; Qu et al., 2019).

**Figure 2:**
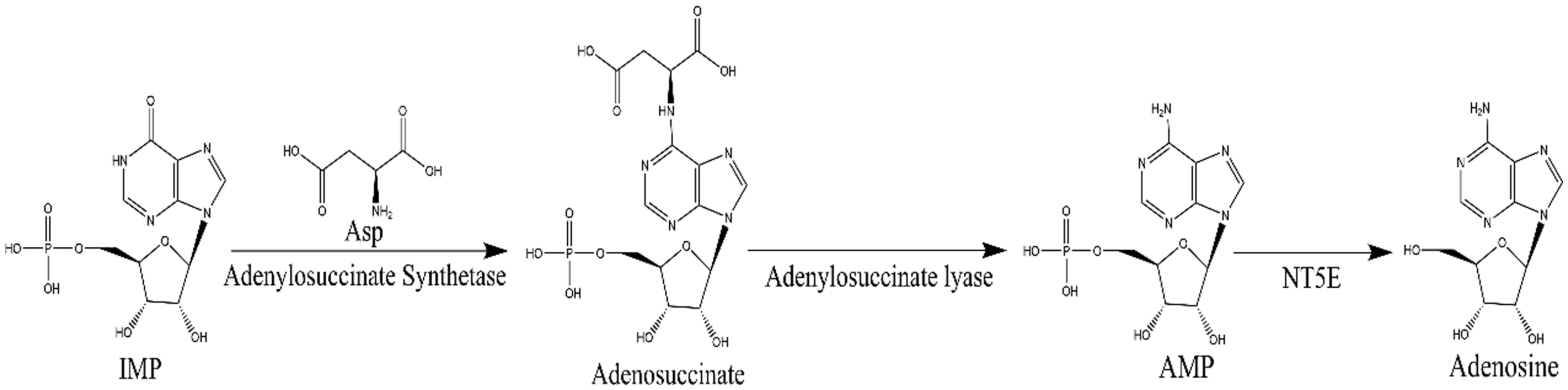
The biosynthetic pathway of adenosine: inosinate acid (IMP) and aspartic acid in the presence of adenylosuccinate synthetase to produce adenosuccinate, which in the further generates fumaric acid and AMP by adenylosuccinate lase, then AMP by 5′-nucleotidase (NT5E) to generate adenosine.

N^6^-(2-hydroxyethyl) adenosine (HEA) is an important bioactive substance with cancer cell inhibition (Xie et al., 2020; Chen et al., 2021; Fu et al., 2023), kidney protection (Wang et al., 2019; Deng et al., 2020), and anti-inflammatory effects (Lu et al., 2015). Therefore, it has attracted much attention for its potential application in the medical industry, especially in the field of cancer therapy. Currently, HEA has been obtained mainly by chemical synthesis (Qu et al., 2006) and liquid fermentation mycelium extraction (Qu et al., 2006). However, chemical synthesis of HEA involves multiple steps, low yields and expensive raw materials, hindering its synthesis on an industrial scale. The liquid fermentation process is easy to control and has become the main means of HEA production (Tsai et al., 2021). It is undoubted that the disclosure of HEA biosynthesis pathway will open up new ways for the wider application of HEA. Previously, a many of generalized Cordyceps species and the strains isolated from it had been characterized as the main HEA-producing species, such as *Cordyceps cicadae* (Zhang et al., 2018), *Cordyceps militaris* (Zhang et al., 2016), *Beauveria bassiana* (Liu et al., 2017), *Cordyceps pruinose* (Meng et al., 2015a, 2015b) *Isaria tenuipes* (Chang et al., 2019) etc. It was demonstrated that A diverse range of amino acids promoted the production of HEA in *Isaria tenuipes* E3, such as arginine, L-glutamic acid, and serine etc., among which arginine is reported as the most effective amino acid (Lei et al., 2013). Moreover, the supplementation of serine, alanine, histidine, and aspartic acid to the culture medium can promote the increase of mycelial HEA content. Three precursor substances, including hypoxanthine, adenine, and adenosine, which share similar chemical structure to HEA, can increase the content of HEA in the mycelium during fermentation (Lei et al., 2014). By comparing the chemical structures of adenosine and HEA, it is seen that HEA has an additional hydroxyethyl group at the N6 position of the purine ring of adenosine, meaning that the HEA is an analog of adenosine. Recently, the biosynthetic pathway of adenosine in *Cordyceps militaris* has been resolved as is shown in Figure 2. IMP and aspartic acid are converted into adenosuccinate under the catalysis of adenylosuccinate synthase, which is successively catalyzed by adenylosuccinate lyase (ADSL) and 5′-nucleotidase (NT5E) to produce adenosine. Given the structural similarity between HEA and adenosine, it is speculated that amino acids may be the substrates for HEA synthesis.

In recent years, based on the advances of multi-omics technologies, especially metabolomics and transcriptomics, has been reported in illuminating the biosynthesis of active substances, which has provided effective methods for uncovering the biosynthetic pathways of secondary metabolism and identifying candidate genes involved (Han et al., 2016). For example, a previous study on *C. kyushuensis Kob* identified the highly homologous cordycepin biosynthesis gene cluster ck1-ck4 by means of transcriptomic and proteomic analysis (Zhao et al., 2019). Recent studies have uncovered the inherited basis for the development of *C. cicadae*. However, comparative transcriptomic studies related to HEA biosynthesis have not been reported. In this study, due to HEA as a derivative of nitrogen metabolism, we attempted to investigate the effect of nitrogen sources and the means of fermentation on promoting the production of HEA and adenosine, which supplies the evidences for the enclosure of the biosynthetic pathways of HEA.

## 2 Materials and methods

### 2.1 Preparation of *Cordyceps cicadae* strain AH 10-4

The *C. cicadae* strain AH 10-4 with higher yield of HEA was used in this study, which was isolated and purified in our laboratory in the previous study (Huang et al., 2021). *Cordyceps cicadae* stain AH 10-4 was plated to PDA medium and incubated at 26°C for 7 days. Three 25 mm^2^ PDA cultures were punched out by the inoculation spatula and then transferred to the seed medium. The seed culture was then grown in a 250-mL flask containing 100 mL of its medium (per liter: glucose 20 g, peptone 10 g, yeast extract 10 g, KH_2_PO_4_ 2 g, MgSO_4_ 0.5 g, ZnSO_4_ 0.5 g, and pH natural) at 26°C and 140 rpm for 2 days (Huang et al., 2021).

### 2.2 Shaker fermentation

The *C. cicadae* stain AH 10-4 was cultivated in 250-mL flasks containing 100 mL medium using 5% inoculum (v/v) and then cultured at 26°C and 140 rpm for 10 days. The flask samples were harvested every day. The fermentation medium was referenced to the seed medium (increasing the concentration of glucose to 30 g/L).

N^6^-(2-hydroxyethyl) adenosine, as an important nucleoside analog of fungi, was closely linked to nitrogen metabolism. In order to investigate the effects of compound and simple nitrogen sources on the production of adenosine and HEA, compound nitrogen sources (peptone, yeast extract) and simple nitrogen sources (ammonium sulfate, ammonium citrate dibasic, diammonium oxalate monohydrate, and ammonium citrate tribasic) were added to basal medium at 10 g/L supplementation, respectively, and incubated at 26°C and 140 rpm for 5 days. The composition of the basal medium was as following (per liter): glucose 30 g, KH_2_PO_4_ 2 g, MgSO_4_ 0.5 g, ZnSO_4_ 0.5 g, and pH natural.

To investigate the impact of predictive amino acids on HEA production, 30 g/L glucose with 0.25 g/L amino acids were added to 67 g/L yeast nitrogen base w/o amino acids and then cultured at 26°C and 140 rpm for 7 days, the amount of each ingredient added in Table 1. The 15 amino acids are valine, leucine, isoleucine, proline, phenylalanine, tyrosine, tryptophan, threonine, cysteine, methionine, asparagine, glutamine, lysine, arginine, and histidine. The five forecast amino acids are alanine, serine, glutamic acid, aspartic acid and glycine.

**Table 1:** Additive ratio of each ingredient.

Glucose	Yeast nitrogen base w/o amino acids	Amino acid
3%	6.7%	15 + Ala
3%	6.7%	15 + Ser
3%	6.7%	15 + Glu
3%	6.7%	15 + Asp
3%	6.7%	15 + Gly
3%	6.7%	15 + Ala+Ser + Glu + Asp+Gly
3%	6.7%	15
3%	6.7%	Control

“15” represents 15 amino acids, including valine, leucine, isoleucine, proline, phenylalanine, tyrosine, tryptophan, threonine, cysteine, methionine, asparagine, glutamine, lysine, arginine, and histidine.

### 2.3 Stationary fermentation

The *C. cicadae* stain AH 10–4 was cultivated in 250-mL flasks containing 100 mL medium using 5% inoculum (v /v). It was cultured at 26°C for 17 days by liquid static culture. In order to investigate the effect of amino acids on the production of HEA, amino acids (alanine, serine, aspartic acid, glycine as well as glutamic acid), were added at an addition of 5 g/L to the base medium (per liter: glucose 30 g, peptone 5 g, KH_2_PO_4_ 2 g, MgSO_4_ 0.5 g, ZnSO_4_ 0.5 g, pH natural). The incubation was then incubated at 26°C for 17 days.

### 2.4 Submerged fermentation

Submerged fermentation was cultured with a 5% seed-cultured solution at 26°C, shaking at 140 rpm in the first 3 days and then static incubation for the latter 6 days. It was collected after 9 days incubation.

### 2.5 Extraction and quantitative analysis of adenosine and HEA

The fermentation broth of *C. cicadae* strain AH 10-4 was centrifuged at 4,500 rpm for 30 min to obtain mycelium, which was then oven-dried at 60°C to a constant weight. For the extraction of adenosine and HEA, the mycelium was ground into a powder that was sieved through a 60-mesh sieve. Then the powder was weighed and extracted by ultrasonic extraction with 20 fold of ultrapure water for 90 min with mixing at 10 min intervals. The extracts were centrifuged at 4,500 rpm for 30 min, and the supernatant was filtered through a Minisart R filter with a pore size of 0.22 μm for later use. The HPLC detection conditions for adenosine and HEA were consistent with the previous studies (Huang et al., 2021). The adenosine and HEA content in mycelium was measured in mg/g, with “g” representing the dry weight of the mycelium.

### 2.6 RNA extraction and transcriptomic sequencing

Total RNA was extracted from mycelium in shaker and stationary cultures (ammonium sulfate as nitrogen source) by the TRIzol method. The poly-T Oligo-attached magnet was used to enrich mRNA from the total RNA, which was subsequently fragmented in fragmentation buffer. And then a total amount of 1.5 μg mRNA was used to construct cDNA library for each sample by using Illumina’s NEBNext® Ultra™ RNA Library Preparation Kit. 200–250 bp cDNA fragments were screened and purified using the AMPure XP system (Beckman Coulter, Beverly, United States). The quality of the libraries was then assessed on an Agilent Bioanalyzer 2100 system and sequenced (150 bp paired-end) on an Illumina Novaseq 6000 platform. A total of six libraries were constructed with three biological replicates per sample.

### 2.7 *De novo* assembly and annotation

The clean reads were obtained by removing reads with adapter, ploy-N, and low quality reads from the raw paired-end reads, which were then assembled using Trinity (Grabherr et al., 2011). To further explore the annotation function of the assembled and spliced transcripts, the assembled unigene was annotated against Nt, Nr, Swiss-Prot, GO, KOG, KEGG, and Pfam databases to obtain the annotation of the unigene.

### 2.8 Differential expression analysis and functional enrichment

The stationary and shaker fermentation mycelium was analyzed for differential expression using the DESeq R software package (Anders and Huber, 2010). The genes with a *p* value of less than 0.05 on an adjusted basis found by DESeq were then classified as differentially expressed genes. In addition, the mycelium from stationary and shaker fermentation cultures were analyzed for functional enrichment that was performed for GO and KEGG by using the GOsep R software package and KOBAS, respectively (Mao et al., 2005). The screening criteria for significantly enriched GO and KEGG by DEGS were Bonferroni corrected *p* values of less than or equal to 0.05.

### 2.9 Statistics

The data were reported as mean ± SD and analyzed using one-way ANOVA with Duncan’s multiple rang test in SPSS software version 23.0 (IBM Corp., Armonk, NY, United States) to determine the significance of group differences. A *p* value less than 0.05 was considered statistically significant.

## 3 Results

### 3.1 Production of HEA in the *Cordyceps cicadae* strain AH 10-4 by shaker culture

**Figure 3:**
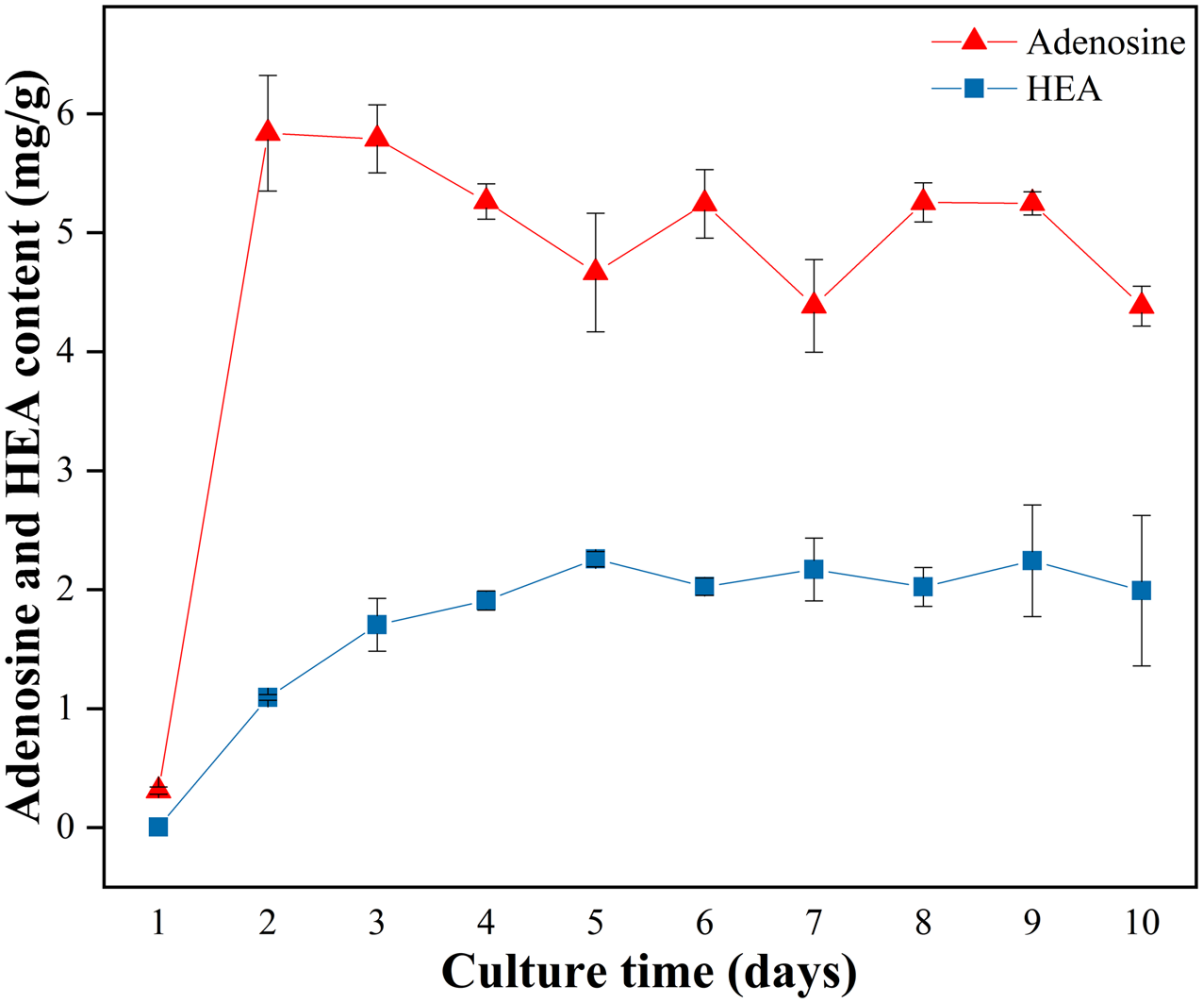
Production of adenosine and HEA within strain AH 10-4 mycelial during shaker culture. Values are shown as mean ± SD of triple determinations.

A time course analysis was conducted to study the kinetics of HEA production in strain AH 10–4 grown in shaker culture for 10 days. Firstly, in terms of biomass, it was seen that the biomass of mycelium increased with incubation time from Day 1 to Day 8, reaching the highest level of 2.403 g/100 mL on Day 8, and then slightly decreased (Supplementary Table S1). As was shown in Figure 3, after the 2 days culture, the content of adenosine reached to the highest level of 5.836 ± 0.486 mg/g, and then decreased gradually until Day 5. Distinct from adenosine, the content of HEA gradually increased with the incubation time, reaching its highest level on the 5th day (2.257 mg/g), and then slightly decreased since the sixth day. Interestingly, the content of HEA obviously increased from Day 2 to Day 5, in which time the content of adenosine decreased gradually, showing a correlation between the increasing trend in HEA production and the decreasing trend in adenosine production. Therefore, we speculated that there was a connection between the synthesis pathways of HEA and adenosine.

### 3.2 Effect of different nitrogen sources on the production of HEA by shaker culture

**Figure 4:**
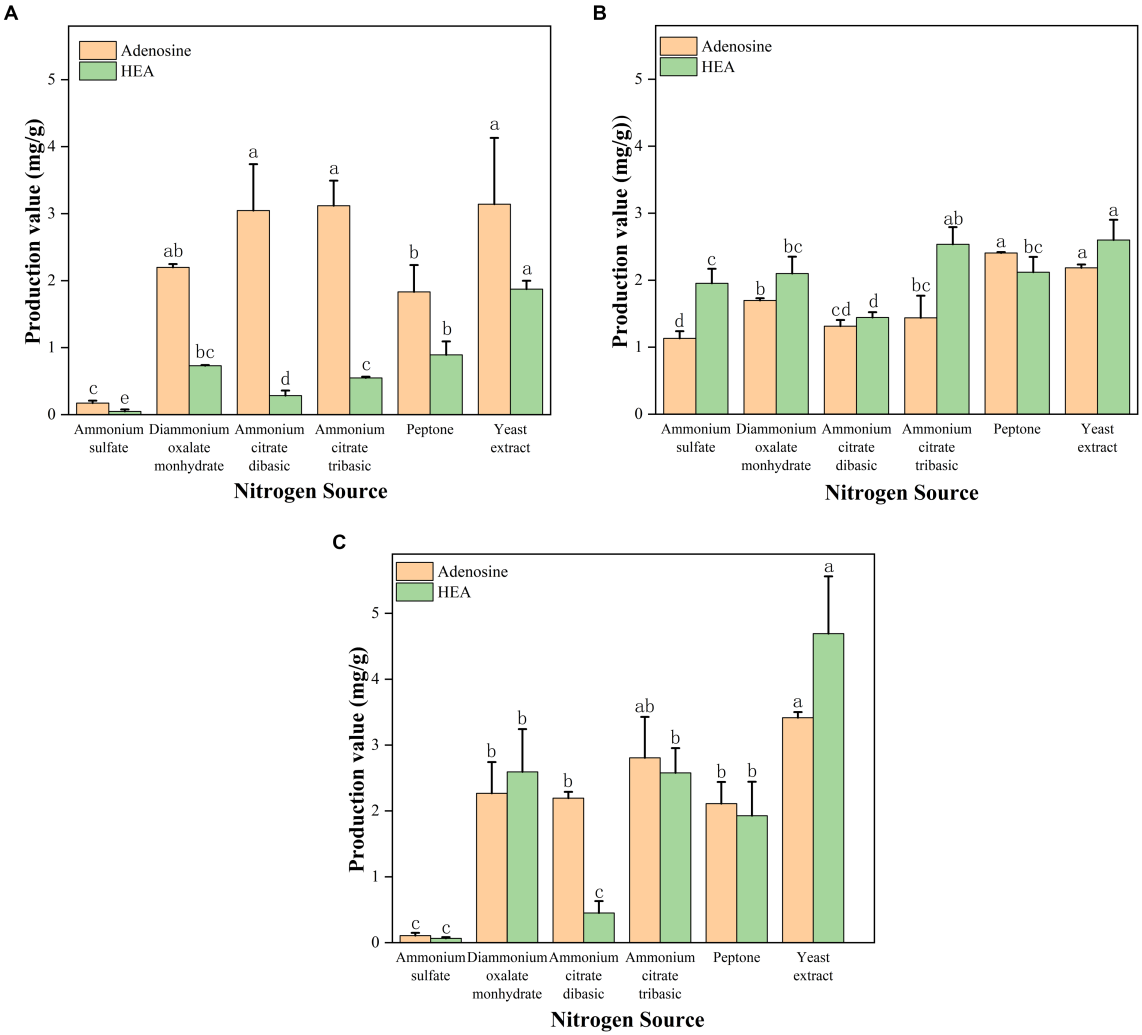
**(A)** Effect of shaker culture with different nitrogen source on the production of adenosine and HEA. **(B)** Effect of stationary culture with different nitrogen source on the production of adenosine and HEA. **(C)** Effect of submerged fermentation with different nitrogen source on the production of adenosine and HEA. Values are shown as mean ± SD of triple determinations. Different letters indicate significantly different values according to a one-way ANOVA with Duncan’s multiple test (*p* < 0.05).

Due to the reason that adenosine and HEA belong to nucleosides produced by fungus, and nucleosides are known to be the derivatives of nitrogen metabolism. Given the advantages of the shaker culture, such as shorter fermentation time, it was chosen as the main method for obtaining mycelium. Thus, in order to investigate the effects of various nitrogen sources on the content of adenosine and HEA in mycelial, we measured the production of adenosine and HEA after 5 days of shaker culture, a time point that showed optimal of HEA production in Figure 3. As shown in Figure 4A, the HEA content was 0.047, 0.730, 0.284, and 0.547 mg/g mycelia dry weight by using ammonium sulfate, diammonium oxalate monohydrate, ammonium citrate dibasic, and ammonium citrate tribasic as nitrogen source, respectively. Among the ammonium salts, the use of diammonium oxalate monohydrate and ammonium citrate tribasic possessed more superior HEA content in mycelium, while the biomass of ammonium citrate tribasic was remarkably higher than that of diammonium oxalate monohydrate (*p* < 0.05) (Supplementary Table S2). Therefore, ammonium citrate tribasic was determined to be the optimum nitrogen source among the ammonium salts. Distinct from ammonium salts, the yields of HEA were 1.872 and 0.892 mg/g from yeast extract and peptone medium respectively, which were significantly superior to those from ammonium salts (*p* < 0.05). By comparison with ammonium salts, complex nitrogen sources (peptone, yeast extract) were more favorable for the accumulation of HEA production in mycelium during shaker fermentation, which might due to their enrichment in amino acids.

### 3.3 Effect of different nitrogen sources under stationary fermentation on the production of HEA

In contrast to shaker fermentation, it has been demonstrated that stationary culture has the advantages of low energy consumption, low requirements for production conditions and easy scale-up (Liao et al., 2021). Therefore, we probed the ability of various nitrogen sources to yield HEA and adenosine after 17 days of stationary culture. In terms of biomass, complex nitrogen sources (yeast extract, peptone) promoted the growth of *C. cicadae* strain AH 10-4 mycelium better than ammonium salt (*p* < 0.05) (Supplementary Table S3), including ammonium citrate tribasic, ammonium citrate dibasic, and diammonium oxalate monohydrate (Figure 4B). Interestingly, ammonium citrate tribasic, ammonium citrate dibasic, and diammonium oxalate monohydrate were better than ammonium sulfate in stimulating mycelial growth of *C. cicadae (p < 0.05)*. Complex organic nitrogen sources (yeast extract, peptone) were found to favor adenosine accumulation in the mycelium. As for HEA production, yeast extract and ammonium citrate tribasic were the most effective nitrogen sources, with HEA yields of 2.599 and 2.535 mg/g, respectively. These were followed by diammonium oxalate monohydrate and peptone (*p* < 0.05). Notably, stationary fermentation caused an increase in the HEA content within mycelium, while the opposite was found for the biomass. Therefore, diammonium oxalate monohydrate and ammonium citrate tribasic can be considered viable alternatives to complex nitrogen source to obtain high HEA yield industrially through stationary fermentation.

### 3.4 Effect of different nitrogen sources on the production of HEA by submerged fermentation

Submerged fermentation, a novel approach combining shaker and stationary fermentation, was investigated in order to identify an optimal fermentation method and nitrogen source for industrial application. We also explored the ability of different nitrogen sources to produce HEA of *C. cicadae* strain AH 10-4 under submerged fermentation. The result of mycelial biomass was shown in Figure 4C. Yeast extract was most effective for the growth of *C. cicadae* mycelium, which was followed at more than 1 g/100 mL for ammonium citrate, diammonium oxalate monohydrate and peptone as nitrogen source (Supplementary Table S4). Yeast extract as a nitrogen source yielded the highest levels of adenosine (3.416 mg/g) and HEA (4.691 mg/g) within *C. cicadae* strain AH 10–4 mycelium. The HEA concentration was 1.928, 0.065, 2.593, 0.450, and 2.578 mg/g mycelia dry weight by using peptone, ammonium sulfate, diammonium oxalate monohydrate, ammonium citrate dibasic, and ammonium citrate tribasic. The result indicated that diammonium oxalate monohydrate and ammonium citrate tribasic were comparable to peptone in enhancing HEA accumulation within the mycelium. Additionally, ammonium citrate tribasic and diammonium oxalate monohydrate were inexpensive and could be used as an alternative source of organic nitrogen. Notably, ammonium citrate tribasic had superior production of adenosine and HEA compared to diammonium oxalate monohydrate. Consequently, ammonium citrate tribasic was regarded as the optimal nitrogen source to produce higher HEA concentrations in *C. cicadae* strain AH 10-4 mycelium. Submerged fermentation increased the biomass and reduced incubation time than stationary fermentation. Importantly, this can be easily scaled-up in the fermentation industry.

### 3.5 Illumina sequencing and *de novo* assembly

In this study, we did transcriptomic analysis to compare mycelium under high-HEA-producing and low-HEA-producing conditions with the aim to find the different metabolic pathways related to HEA biosynthesis. Comparing HEA content in stationary (Figure 4B) and shaker cultured mycelium (Figure 4A), ammonium sulfate has a simple composition and a significant difference of HEA content, which was chosen a suitable nitrogen source for transcriptomics. Therefore, samples of mycelium cultured with ammonium sulfate as a nitrogen source in stationary (A1-3) and shaker (B1-3) cultures, including three biological replicates, were selected for transcriptomics. For the purpose of distinguishing transcriptomic features during HEA biosynthesis, RNA-seq libraries were performed by extracting total RNA from stationary and shaker fermentation mycelium, respectively. Then, the RNA-seq libraries were sequenced using Illumina Novaseq 6000. The raw reads obtained for the stationary culture and shaker fermentation samples ranged from 50,645,198 to 59,883,168, and raw reads were filtered to obtain clean reads ranging from 50,041,026 to 59,299,218 (Table 2). Among all six samples, the proportion of bases with quality value ≥20 was above 97.58% in Q20 and the proportion of bases with quality value ≥30 was above 93.55% in Q30 in all groups, which indicated that the sequencing results were satisfactory and could be used for subsequent analysis.

**Table 2:** Overview of transcriptome sequencing and *de novo* assembly results.

Sample	Raw reads	Clean reads	Error rate	Q20	Q30	GC content
A_1	58,062,016	57,581,622	0.03%	97.58%	93.55%	56.12%
A_2	50,645,198	50,041,026	0.03%	97.68%	93.69%	55.80%
A_3	59,883,168	59,299,218	0.02%	97.94%	94.46%	55.53%
B_1	56,441,966	55,835,776	0.03%	97.70%	93.91%	55.73%
B_2	53,825,912	53,189,416	0.03%	97.68%	93.83%	55.60%
B_3	57,562,606	56,878,932	0.03%	97.68%	93.92%	55.94%

**Figure 5:**
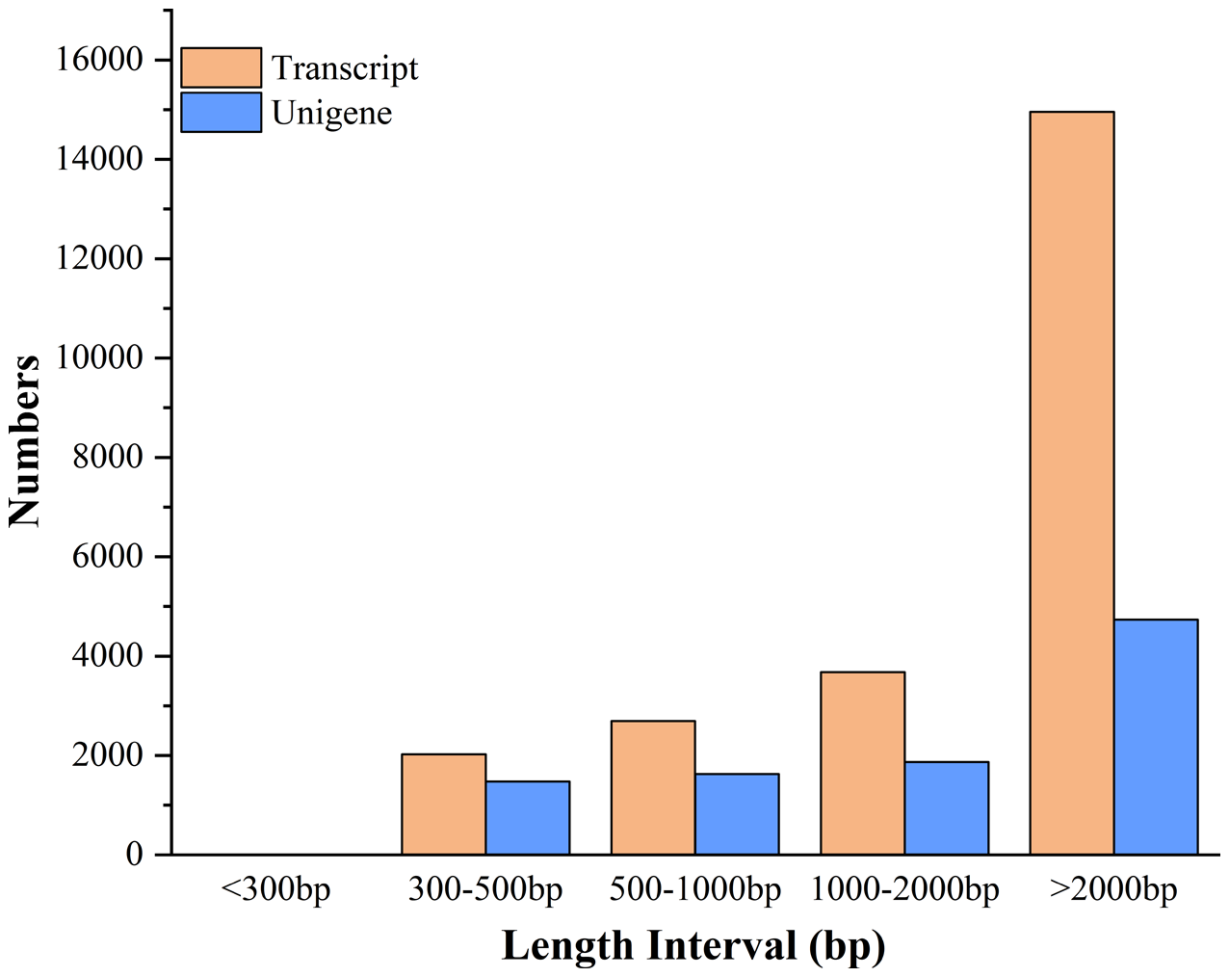
Length distribution of assembled transcripts and unigenes between stationary and shaker fermentation mycelium.

The clean reads were assembled into 9,713 unigenes and 23,356 transcripts using Trinity software (Trinity Technologies, CA, United States), with an average assembled single gene length of 2,721 bp (N50 = 4,483) (Supplementary Table S5). The 23,356 transcripts comprised 2,024 (8.67%) transcripts within the length range of 300–500 bp, 2,694 (11.53%) with 500–1,000 bp, 3,680 (15.76%) with 1,000–2,000 bp, and 14,958 (64.04%) with >2,000 bp. The 9,713 unigenes consist of 1,478 (15.22%) unigenes with length range of 300–500 bp, 1,627 (16.75%) with 500–1,000 bp, 1,872 (19.27%) with 1,000–2,000 bp, and 4,736(48.76%) with the length of more than 2,000 bp (Figure 5).

**Figure 6:**
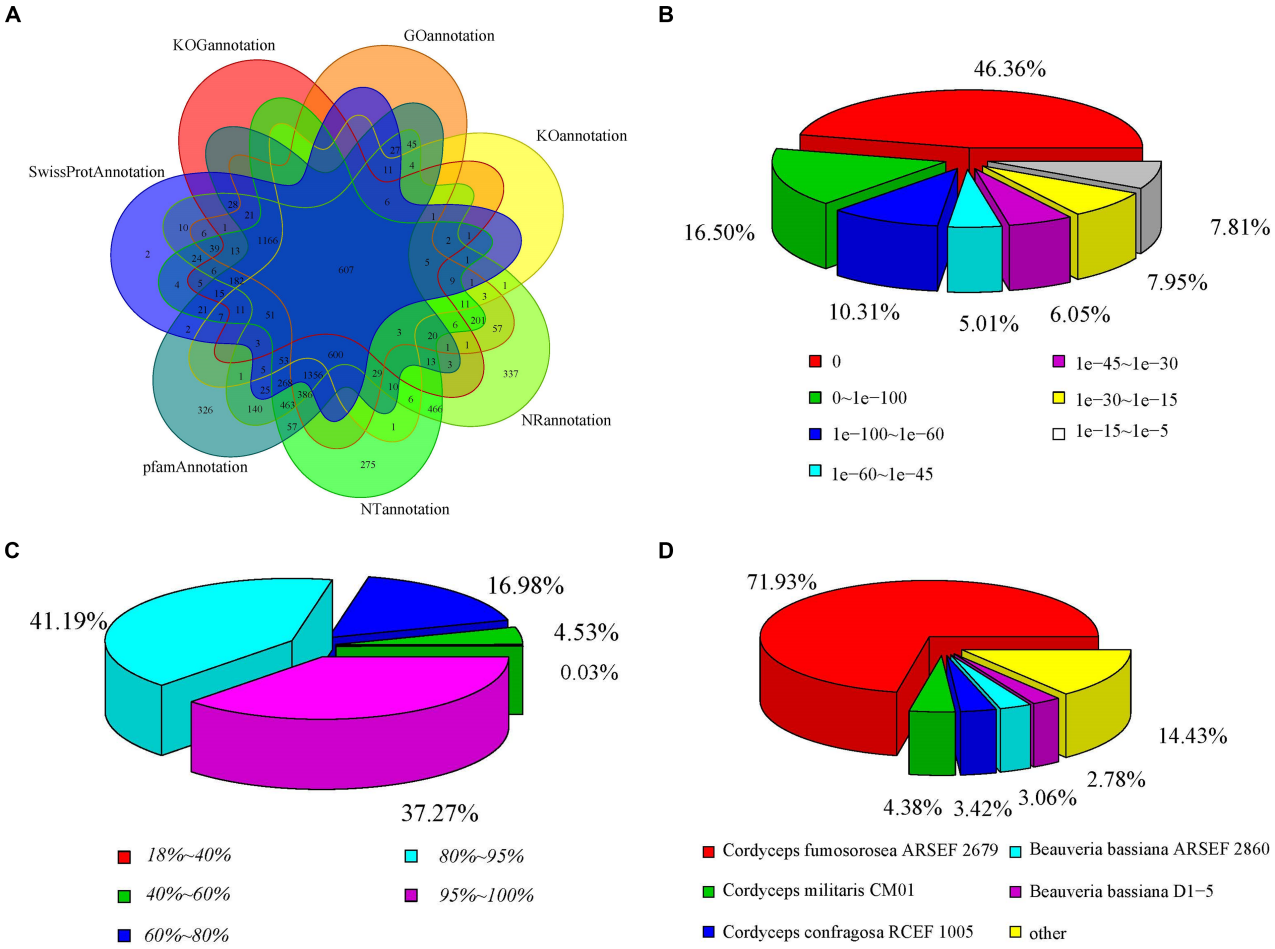
Distribution of the homology search against the NCBI database between stationary and shaker fermentation mycelium: **(A)** Venn diagram of the number of orthologous unigenes. **(B)** Distribution of *E*-value. **(C)** Similarity of expressed sequence tags against the NR database. **(D)** Distribution of annotated species.

To annotate the gene functions of the stationary and shaker culture samples, the Nr, Nt, Pfam, KOG, Swiss-Prot, KEGG, and GO databases were used, which matched to 6,734, 6,393, 5,966, 2,171, 4,595, 1,443, and 4,636 unigenes, respectively, (Supplementary Table S6). The annotations of the unigenes sequences in the seven database species were shown in the Figure 6A, which indicated that the unigenes sequences were quite similar to the known proteins in the public databases. The 46.36% of the genes displayed an *E*-value of 0, indicating the reliability of these matches (Figure 6B). Regarding the identity distribution of the predicted proteins, the majority of hits (78.46%) in the NR database exhibited 80–100% identity with other fungi proteins, while only 0.03% of the sequences had 18–40% identity (Figure 6C). Furthermore, the distribution of species was also analyzed (Figure 6D), with the annotation results based on the NR library indicating that the genes of *C. cicadae* displayed the highest number of matches with the genes of *Cordyceps fumosorosea*.

### 3.6 DEGs between stationary and shaker fermentation mycelial libraries

Approximately 87–89% of the total reads were successfully mapped to the *C. cicadae* transcriptome (Supplementary Table S7), indicating a high mapping efficiency. We then examined the number of DEGs, with *p* value of less than or equal to 0.05 (Supplementary Figure S3A). A total of 4,121 DEGs were detected, with 2,038 upregulated and 2,083 downregulated genes (Supplementary Figure S3B). The Venn diagram analysis revealed that 6,499 genes were co-expressed genes (Supplementary Figure S4). These data indicated that the gene expression patterns remained consistent in mycelium cultured with ammonium sulfate as nitrogen source in stationary and shaker cultures.

### 3.7 Functional and GO annotations of DEGs

**Figure 7:**
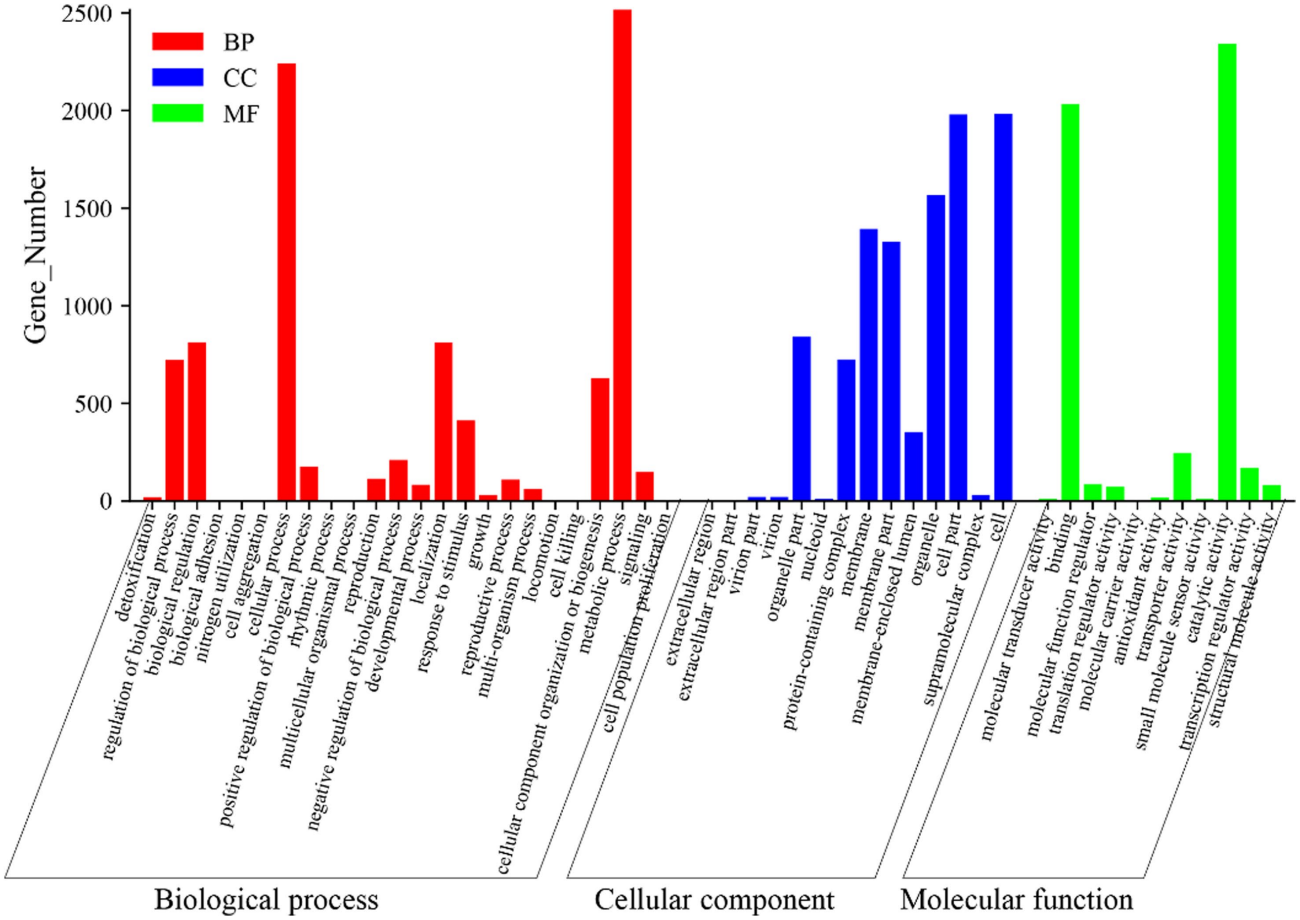
GO analysis between stationary and shaker fermentation mycelium. BP, Biological processes; CC, Cellular components; and MF, Molecular functions.

To explore the functions of the identified unigenes in the *C. cicadae* transcriptome analysis, GO enrichment analysis was performed. The successfully annotated unigenes were grouped according to GO categories, including biological processes (BP), cellular components (CC) as well as molecular functions (MF). It was found that 9,713 unigenes were classified into 49 functional groups. The most common of these groups were metabolic process (2,516 unigenes), cell (1,984 unigenes), and catalytic activity (2,342 unigenes) (Figure 7). This analysis provided insights into the diverse functions and activities of the identified unigenes in *C. cicadae*.

**Figure 8:**
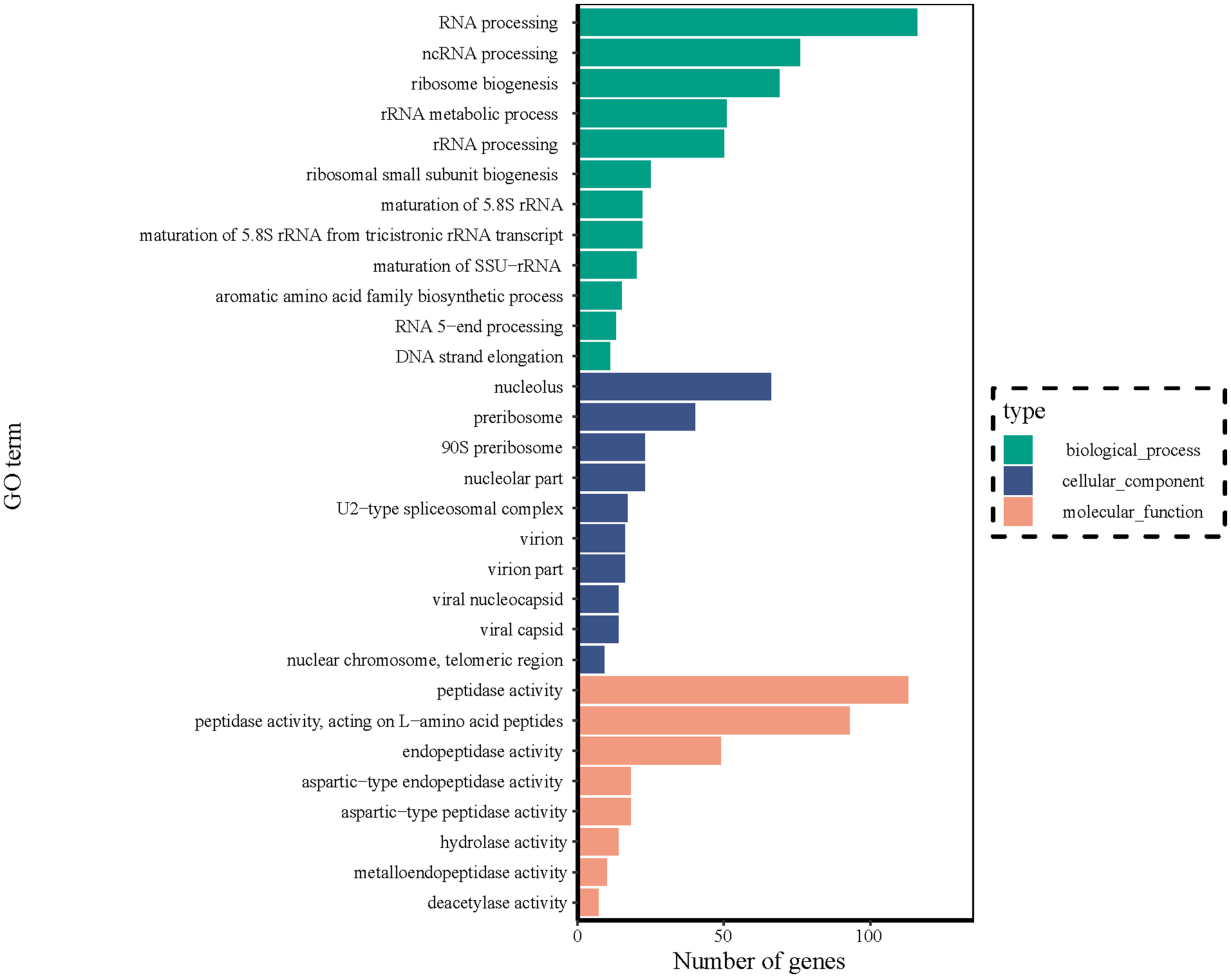
DEGs of transcripts in different GO categories between stationary and shaker fermentation mycelium. BP, Biological processes; CC, Cellular component; and MF, Molecular function.

The DEGs identified between stationary and shaker fermentation were further used for GO enrichment analysis. It was found that the DEGs were mainly related to “RNA processing” (116), “ncRNA processing” (76), “nucleolus” (66), “ribosome biogenesis” (69), “preribosome” (40), “peptidase activity” (113), “peptidase activity, and acting on L-amino acid peptides” (93) (Figure 8). These up- and downregulated genes were crucial for the notable difference in HEA production between stationary and shaker-cultured mycelium. Thus, these DEGs provided potential targets for future functional studies aiming to elucidate the underlying mechanisms involved in HEA production.

### 3.8 Mapping and enrichment of KEGG pathways

**Figure 9:**
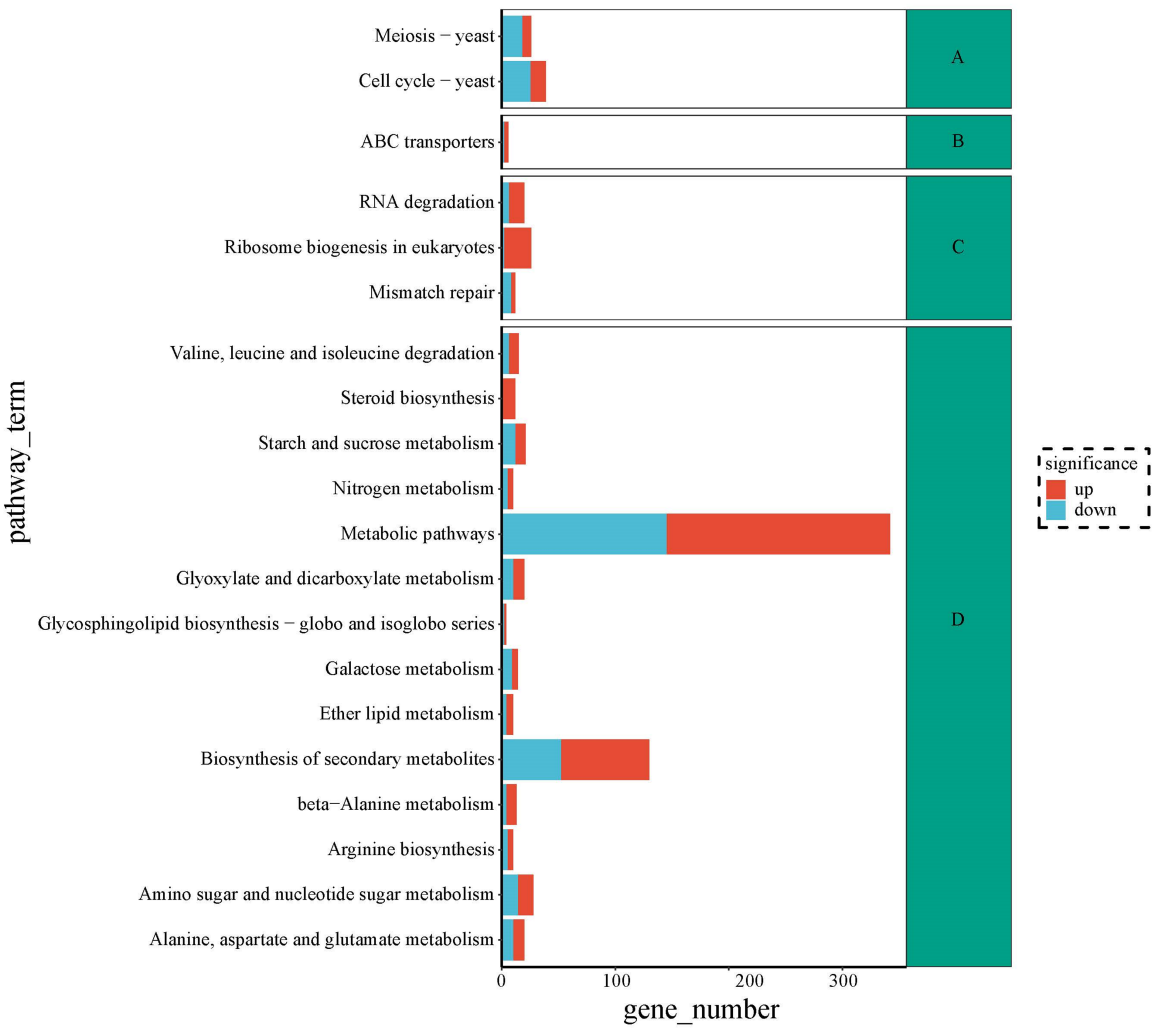
The top 20 metabolic pathways between stationary and shaker fermentation mycelium: **(A)** Cellular processes; **(B)** Environmental information processing; **(C)** Information processing; **(D)** Metabolism.

To seek metabolic pathways related to HEA synthesis, we annotated all differentially expressed genes against the KEGG database. The DEGs of top 20 pathways were categorized into four branches: cellular processes, environmental information processing, information processing and metabolism. Among the top 20 pathways, the highest number of differential genes are shown in Figure 9. The largest group of the top 20 enriched pathways was responsible for metabolism, including “Alanine, aspartate and glutamate metabolism” (20), “Glyoxylate and dicarboxylate metabolism” (20), “Galactose metabolism” (14), “Nitrogen metabolism” (10), “Starch and sucrose metabolism” (21), “Amino sugar and nucleotide sugar metabolism” (28), ‘beta-Alanine metabolism” (13), “Ether lipid metabolism” (10), “Biosynthesis of secondary metabolites” (130), “Glycosphingolipid biosynthesis—globo and isoglobo series” (4), “Valine, leucine and isoleucine degradation” (15), “Arginine biosynthesis” (10), “Steroid biosynthesis” (12), and “Metabolic pathways” (342). We speculated that HEA synthesis might be closely linked to pathways such as “alanine, aspartate and glutamate metabolism,” “nitrogen metabolism” and “β-alanine metabolism.” It was well known that amino acids provided the backbone of the purine metabolic pathway. However, the relationship of alanine, aspartate and glutamate metabolism to the synthesis of HEA needs to be further investigated. Furthermore, HEA is an adenosine derivative, so transcriptional studies of amino acid synthesis pathways and purine metabolic pathways of metabolism in combination with analyses of biosynthesis genes have the potential to dissect biosynthesis pathways of HEA.

### 3.9 Effect of amino acid type on HEA production

As shown in Figure 4A, the complex nitrogen source promoted the accumulation of HEA in mycelium during shaker fermentation. The complex nitrogen sources had a superior production of HEA since they were rich in amino acids. In addition, alanine, aspartate, and glutamate as well as arginine metabolic pathways were significantly different, which is consistent with our speculation that amino acids can be used as substrates for HEA synthesis (Figure 9). Due to HEA as a purine derivative, glutamic acid, glycine, serine, and aspartic acid are involved in purine metabolism. In addition, the former research has also indicated that alanine promotes HEA accumulation in mycelium (Lei et al., 2014). Therefore, we used yeast nitrogen base w/o amino acids supplemented with 15 amino acids as the basal medium, and then the five presumed amino acids were added, respectively, to explore their relationship with the HEA content. The result was as shown in Table 3, there was no statistically significant difference in the content of HEA between the group with the addition of the 15 amino acids and the control group, so we inferred that the 15 amino acids had no relevance to the biosynthesis of HEA (Table 3). However, the group supplemented with glutamate exhibited significantly higher HEA content compared to the other groups.

**Table 3:** Effect of 20 amino acids on production HEA in mycelium.

Amino acid types	HEA content(mg/g)
15 + Ala	0.020 ± 0.008b
15 + Ser	0.015 ± 0.003b
15 + Glu	0.078 ± 0.064a
15 + Asp	0.015 ± 0.000b
15 + Gly	0.012 ± 0.006b
15 + Ala+Ser + Glu + Asp+Gly	0.027 ± 0.007b
15	0.010 ± 0.000b
Control	——b

“15” represents 15 amino acids, including valine, leucine, isoleucine, proline, phenylalanine, tyrosine, tryptophan, threonine, cysteine, methionine, asparagine, glutamine, lysine, arginine, and histidine. Values are showed as mean ± SD of triple determinations. Different letters in the same column indicate significantly different values according to a one-way ANOVA with Duncan’s multiple test (*p* < 0.05).

**Figure 10:**
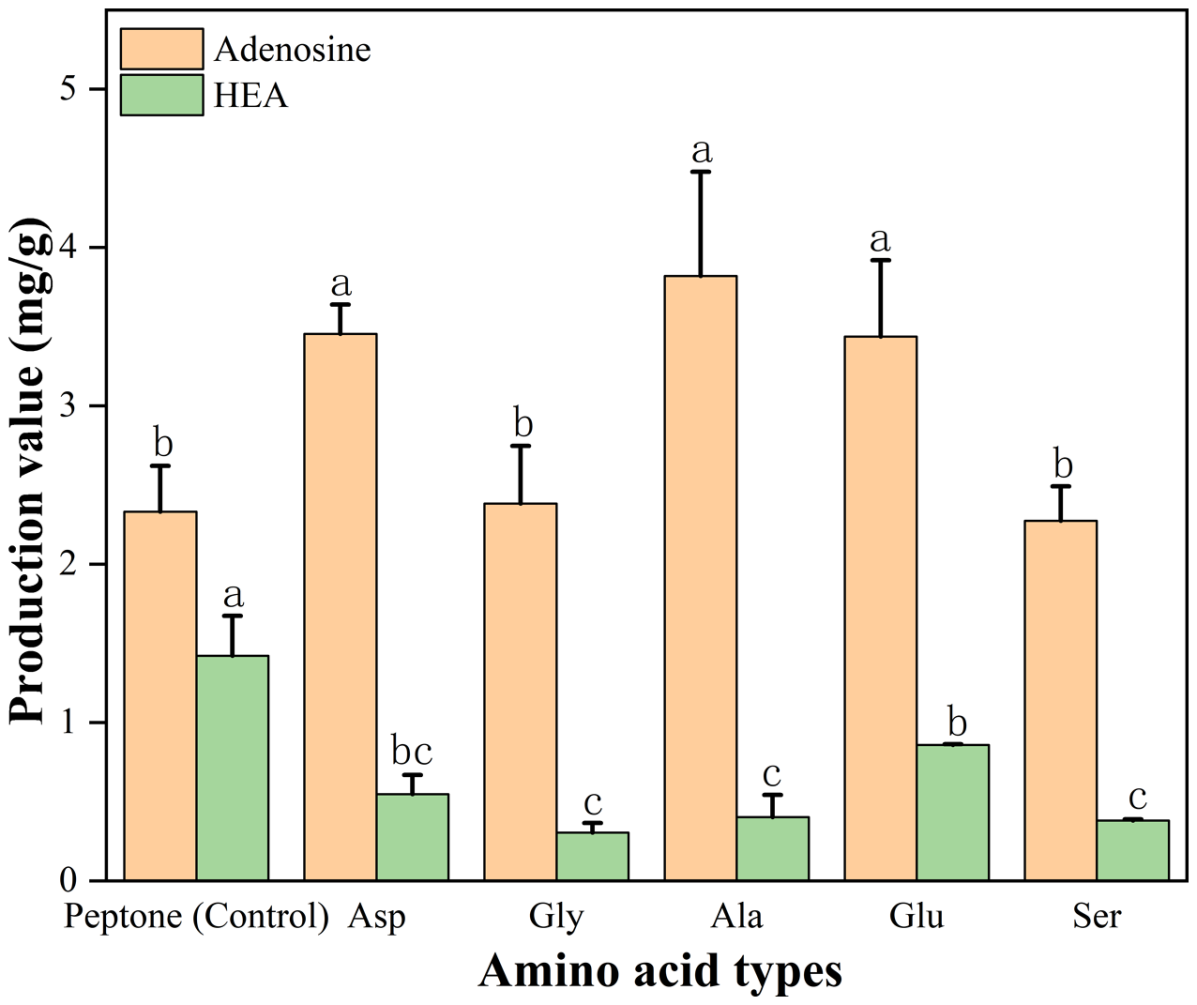
Effect of amino acid type on HEA production in mycelium. Values are showed as mean ± SD of triple determinations. Different letters indicate significantly different values according to a one-way ANOVA with Duncan’s multiple test (*p* < 0.05).

In addition, in order to further validate, we also added the presumed 5 amino acids (Gly, Glu, Ser, Asp, and Ala) to the peptone medium and then HEA content was tested, respectively, after 17 days of stationary fermentation, respectively. Addition of amino acids (glutamate, glycine, serine, aspartate, and alanine) resulted in lower biomass compared to the control (Supplementary Table S8). As shown in Figure 10. When peptone was used as nitrogen source, the adenosine content of the strain AH 10-4 fermenting mycelium was 2.332 mg/g and HEA content was 1.422 mg/g. Interestingly, the addition of amino acids to based medium led to a decrease in HEA content. Hence, it was speculated that excess nitrogen source inhibited HEA synthesis after 17 days of fermentation. Among the peptone medium added with five amino acids, glutamate showed a significant effect on the HEA content in the fermenting mycelia of the strain AH 10-4, which is consistent with the results in Table 3.

## 4 Discussion

In this study, we comprehensively compared the effects of three fermentation methods on HEA production in the *C. cicadae* strain AH 10-4 mycelia. The results revealed that shaker fermentation resulted in high mycelial biomass but low HEA production. Conversely, stationary fermentation favored HEA concentration while exhibiting relatively low biomass even after long-term fermentation. Submerged fermentation, combining with the advantages of both, shortened the incubation time and simultaneously promoted the HEA accumulation. In conclusion, submerged fermentation was deemed more suitable for the industrial-scale HEA production than shaker and stationary fermentation in the *C. cicadae* strain AH 10-4 mycelia, which was consistent with the previous results (Shih et al., 2007). By dissecting the possible factors among these three kinds of fermentation on the perspective of HEA production, dissolved oxygen (DO) was identified as a major factor. To our knowledge, DO was an important indicator for secondary metabolites in the mycelium (Mao and Zhong, 2004; Suparmin et al., 2017), and HEA belonged to the secondary metabolites with special biological activity. Shaker fermentation, with higher DO levels, promoted mycelial growth but inhibited HEA synthesis. On the other hand, the thick mycelia formed by *C. cicadae* in stationary fermentation and submerged fermentation covered the medium surface and seemed to create a hypoxic condition in the medium, which aligned with previous research (Tang and Zhong, 2003). It has been demonstrated that *Cordyceps militaris* induced the production of cordycepin (a nucleoside analog with HEA) under hypoxic conditions (Suparmin et al., 2017), supporting our speculation that high DO inhibits the production of HEA. Transcriptomics results of mycelia from stationary and shaker fermentation with significant up-regulated genes were enriched to TCA and nitrogen metabolism pathways, which might be more favorable for the synthesis of HEA. Another study showed that upregulation of purine metabolism pathway and energy metabolism gene transcript levels promoted the synthesis of precursors, resulting in increased cordycepin content (Long et al., 2023). It indicated that upregulation of energy metabolism genes favored nucleoside synthesis, which was consistent with our findings. Additionally, it has been observed that in some filamentous fungi, the expression of genes in the γ-aminobutyric acid (GABA) shunt pathway is upregulated during hypoxia (Suparmin et al., 2017), redirecting L-glutamate to the TCA cycle and promoting the accumulation of secondary metabolites (Hillmann et al., 2015). Moreover, stationary and submerged fermentations were found more favorable for HEA accumulation production in mycelium might be due to the slow growth of mycelium and glucose metabolism was mainly directed toward HEA synthesis (Zhao et al., 2020).

Fermentation time and cost were considered important limiting factors in the development of the fungal industry. In this study, submerged fermentation achieved a significant reduction in incubation time compared to stationary fermentation, decreasing from 17 days to 9 days, which resulted in a time–cost savings. In summary, submerged fermentation was a more favorable method to produce HEA industrially than shaker or stationary fermentation including three reasons. Firstly, submerged fermentation reduced the fermentation time than stationary fermentation, which resulted in a time–cost savings. Secondly, submerged fermentation needed a lower level of dissolved oxygen. Lower DO promoted the TCA cycle, for which the concentration of HEA in mycelium was favored. Thirdly, biomass was accumulated more in submerged fermentation than stationary fermentation. Biomass served as a crucial indicator of fungal fermentation, and a higher biomass supported the industrial expansion of cultivation.

Nitrogen sources were essential for the growth and nucleoside synthesis regulation of Cordyceps specie (Shih et al., 2007; Su et al., 2021). In our study, *C. cicadae* mycelium had a various preference for nitrogen sources during growth and HEA synthesis. Yeast extract or peptone was rich in nutrients required for growth, providing the necessary foundation for mycelial growth and cell division. Previous studies had shown that complex organic nitrogen sources, such as yeast extract and peptone showed more efficient growth (Kocharin and Wongsa, 2006; Sung et al., 2010, 2011). Interestingly, the ammonium citrate tribasic promoted HEA synthesis than peptone, with HEA production reaching 2.535 and 2.119 mg/g, respectively. The results of our study indicated that the addition of NH_4_^+^ had a direct promotion effect on HEA content. Consistent with previously research, the cordycepin-producing capacity of Cordyceps species can be significantly enhanced by ammonium supplementation (Mao et al., 2005; Leung and Wu, 2007). Furthermore, yeast extract had the most remarkable effect on HEA accumulation. Compared to peptone, yeast extract more effectively promoted HEA synthesis in the mycelium under any fermentation conditions, mainly due to its high utilization by *C. cicadae* for protein, nucleotide synthesis or other substances. As opposed to yeast extract, peptone mainly supported mycelial growth, and then synthesized its own nitrogenous substances. A previous study showed yeast extract was the optimal additional nitrogen source for increasing adenosine and HEA concentrations in PDB (Liang et al., 2014; Ke and Lee, 2019). Considering cost, ammonium citrate tribasic promoted HEA accumulation in the mycelium, following yeast extract with a HEA yield of 2.578 mg/g. Another study showed ammonium citrate tribasic as a major factor in nucleoside (cordycepin) synthesis, which was consistent with our results (Long et al., 2023). Overall, based on the accumulation of HEA using different nitrogen sources under three fermentation conditions, the use of ammonium citrate tribasic provided the possibility of industrialized production of HEA during submerged fermentation.

Furthermore, our research utilized comparative transcriptomics to explore the potential association between amino acids and HEA biosynthesis. By comparing mycelium with high and low HEA production, we identified significant differences in amino acid metabolic pathways, including β-alanine metabolism, arginine biosynthesis as well as alanine, aspartate, and glutamate metabolism, which indicated a robust relationship between amino acids and the biosynthesis of HEA. It was speculated that amino acids might be direct or indirect substrates for HEA synthesis. Previous studies showed that the content of glutamate was higher than other amino acids (Ji et al., 2023; Tang et al., 2023), which may be related to the involvement of glutamate in the synthesis of some critical substance in *C. cicadae*.

Our study showed that glutamate was closely linked to the biosynthetic pathway of HEA. Since HEA as a purine derivative, amino acid synthesis pathway and purine metabolism pathway transcriptional studies as well as gene analyses of HEA biosynthesis may be useful in exploring the biosynthesis pathway of HEA. Transcriptome analysis of *C. cicadae* strain AH 10-4 identified two ADSS sequences, with one showing a significant fold difference of 27.173. Previous research had demonstrated that ADSS was a key enzyme in the adenosine synthesis pathway (Xia et al., 2017). Considering the complexity of the HEA synthesis pathway, we believed that the correlation between ADSS and the HEA synthesis pathway needs to be further verified. The growth kinetics of *C. cicadae* indicated a correlation between adenosine and HEA biosynthesis pathways, which was consistent with previous studies (Liu et al., 2017). Based on this, we proposed the following hypotheses regarding the synthesis pathway of HEA. Firstly, there was a competition between adenosine and HEA synthesis, potentially due to shared precursor substances. Secondly, adenosine consistently exceeded the yield of HEA. This suggested that adenosine might serve as a precursor for HEA synthesis and that other regulatory factors might limit the process of HEA synthesis. In addition, by analyzing the purine metabolic pathway, we observed that adenosine accumulation was converted to inosine by adenosine deaminase. Adenosine deaminase was a significantly up-regulated gene with a fold difference of 1.752. It was hypothesized that the link between adenosine and HEA may be regulated by adenosine deaminase.

In conclusion, the in-depth study of the production mechanism of HEA can provide guidance for future research and industrial application, which will further improve the yield and process efficiency of HEA, promoting the industrialized production and application of HEA.

## 5 Conclusion

In summary, the effect of different nitrogen sources on HEA production in *Cordyceps cicadae* strain AH 10-4 had been explored under different incubation conditions. The production kinetics of HEA and adenosine exhibited a competitive pattern, implicating a potential shared step in their synthesis. The use of ammonium citrate tribasic resulted in higher HEA content (2.578 mg/g mycelia dry weight) in the mycelium, which provided the possibility of scaled-up HEA production in the fermentation industry by submerged fermentation. Comparative *de novo* transcriptomic analysis of *C. cicadae* mycelium between stationary and shaker fermentation (with ammonium sulfate as nitrogen source) revealed amino acid metabolic pathways were significantly different. Glutamate may play a critical substrate for HEA synthesis, which has yet to be subsequently validated. Further studies are needed to elucidate the mechanism by which these amino acids contribute to HEA biosynthesis. Our results provide a foundation for further pharmaceutical and industrial applications of *C. cicadae*, as well as further exploration of the HEA biosynthesis pathway in *C. cicadae*. Future research can combine with genomics technology to explore the gene regulation mechanism, and further use genetic engineering technology to improve the yield and quality of HEA, which promotes the industrialized production and application of HEA.

## Data availability statement

The datasets presented in this study can be found in NCBI SRA repository, accession number PRJNA1075488 (https://www.ncbi.nlm.nih.gov/sra/PRJNA1075488).

## Author contributions

KZ: Formal analysis, Investigation, Methodology, Writing – original draft. HR: Formal analysis, Investigation, Methodology, Supervision, Writing – review & editing. TW: Formal analysis, Methodology, Investigation, Writing – original draft. HZ: Formal analysis, Writing – review & editing. WH: Investigation, Writing – original draft. QS: Investigation, Writing – original draft.
