# Supplementary material for: Exploiting the roles of nitrogen sources for HEA increment in Cordyceps cicadae
Source: Front Microbiol. 2024 May 13;15:1384027. doi: 10.3389/fmicb.2024.1384027 (PMC11129637; doi:10.3389/fmicb.2024.1384027)
Supplement: Supplementary file 1 [file Table_1.DOCX]

Supplementary Material

**Exploiting the roles of nitrogen sources for HEA increment in *Cordyceps cicadae***

# Supplementary Tables

**Table S1** Biomass of *C. cicadae* strain AH 10-4 mycelial during shaker culture

| Culture time  (days) | Biomass  （g/100 mL） |
| --- | --- |
| 1 | 0.27 ± 0.04 |
| 2 | 0.80 ± 0.10 |
| 3 | 1.17 ± 0.08 |
| 4 | 1.48 ± 0.28 |
| 5 | 1.80 ± 0.06 |
| 6 | 1.98 ± 0.05 |
| 7 | 2.26 ± 0.37 |
| 8 | 2.40 ± 0.03 |
| 9 | 2.28 ± 0.09 |
| 10 | 2.14 ± 0.16 |

Values are showed as mean ± SD of triple determinations.

**Table S2** Effect of shaker fermentation with different nitrogen sources on biomass

| Nitrogen Source | Biomass  （g/100 mL） |
| --- | --- |
| Ammonium sulfate | 0.983 ± 0.032 c |
| Diammonium oxalate monhydrate | 1.073 ± 0.081 c |
| Ammonium citrate dibasic | 1.290 ± 0.075 b |
| Ammonium citrate tribasic | 1.513 ± 0.186 a |
| Peptone | 1.660 ± 0.062 a |
| Yeast extract | 1.683 ± 0.047 a |

Values are showed as mean ± SD of triple determinations. Different letters in the same column indicate significantly different values

**Table S3** Effect of stationary culture with different nitrogen source on biomass

| Nitrogen Source | Biomass  （g/100 mL） |
| --- | --- |
| Ammonium sulfate | 0.543 ± 0.214 b |
| Diammonium oxalate monhydrate | 0.740 ± 0.313 ab |
| Ammonium citrate dibasic | 0.917 ± 0.269 ab |
| Ammonium citrate tribasic | 0.823 ± 0.171 ab |
| Peptone | 0.940 ± 0.121 ab |
| Yeast extract | 1.200 ± 0.180 a |

Values are showed as mean ± SD of triple determinations. Different letters in the same column indicate significantly different values

**Table S4** Effect of submerged fermentation with different nitrogen source on biomass

| Nitrogen Source | Biomass  （g/100 mL） |
| --- | --- |
| Ammonium sulfate | 0.440 ± 0.046 d |
| Diammonium oxalate monhydrate | 1.045 ± 0.035 bc |
| Ammonium citrate dibasic | 1.187 ± 0.176 ab |
| Ammonium citrate tribasic | 0.903 ± 0.072 c |
| Peptone | 1.003 ± 0.074 bc |
| Yeast extract | 1.297 ± 0.076 a |

Values are showed as mean ± SD of triple determinations. Different letters in the same column indicate significantly different values

**Table S5**The total reads length was mapped to the *C. cicadae* transcriptome

| - | Shortest | Median | Longest | N50 | N75 | N90 | Total Nucleotides |
| --- | --- | --- | --- | --- | --- | --- | --- |
| Transcript | 301 | 2989 | 23927 | 5810 | 3616 | 2097 | 88687657 |
| Unigene | 301 | 1940 | 23927 | 4483 | 2621 | 1353 | 26435697 |

**Table S6** The genetic annotation statistics of *C. cicadae*

| Database | Number of Unigenes | Percentage (%) |
| --- | --- | --- |
| Annotated in NR | 6734 | 69.33 |
| Annotated in NT | 6393 | 65.82 |
| Annotated in KO | 1443 | 14.86 |
| Annotated in SwissProt | 4595 | 47.31 |
| Annotated in Pfam | 5966 | 61.42 |
| Annotated in GO | 4636 | 47.73 |
| Annotated in COG/KOG | 2171 | 22.35 |
| Annotated in all Databases | 607 | 6.25 |
| Annotated in at least one Database | 7461 | 76.81 |
| Total Unigenes | 9713 | 100 |

**Table S7** The percent of total reads were mapped to the *Cordyceps cicadae* transcriptome

| SAMPLE | Total reads | Total mapped | Percentage |
| --- | --- | --- | --- |
| A_1 | 57581622 | 51558922 | 89.54% |
| A_2 | 50041026 | 44449474 | 88.83% |
| A_3 | 59299218 | 52781305 | 89.01% |
| B_1 | 55835776 | 48711911 | 87.24% |
| B_2 | 53189416 | 46470202 | 87.37% |
| B_3 | 56878932 | 49560429 | 87.13% |

**Table S8** Effect of amino acid type on HEA production in mycelium.

|  | Biomass  (g/100mL) |
| --- | --- |
| (Control) | 0.720±0.014 a |
| Asp | 0.320±0.057 cd |
| Gly | 0.510±0.113 b |
| Ala | 0.265±0.021 d |
| Glu | 0.410±0.014 bc |
| Ser | 0.395±0.021 bcd |

Values are showed as mean ± SD of triple determinations. Different letters in the same column indicate significantly different values

# Supplementary Figures





**Figure S1** Production of adenosine and HEA within strain AH 10-4 mycelial by using diammonium oxalate monohydrate as the nitrogen source during stationary fermentation. Values are shown as mean ± SD of triple determinations.





**Figure S2** Production of adenosine and HEA within strain AH 10-4 mycelial by using yeast extract as the nitrogen source during submerged fermentation. Values are shown as mean ± SD of triple determinations.

(A) (B)


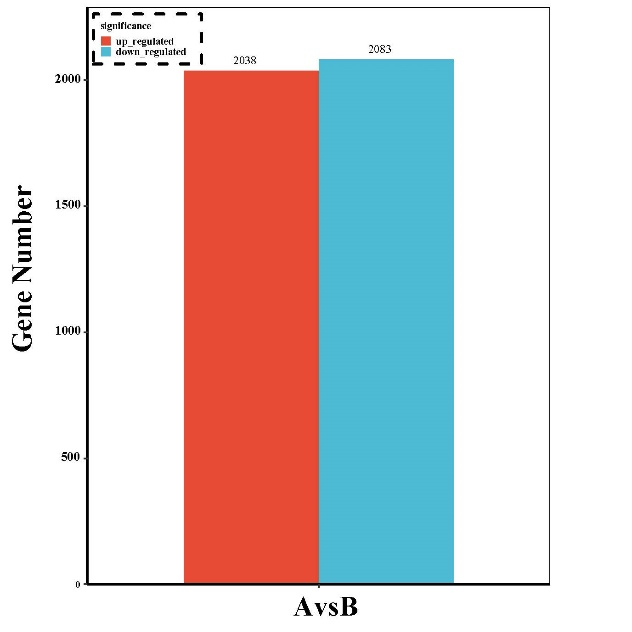

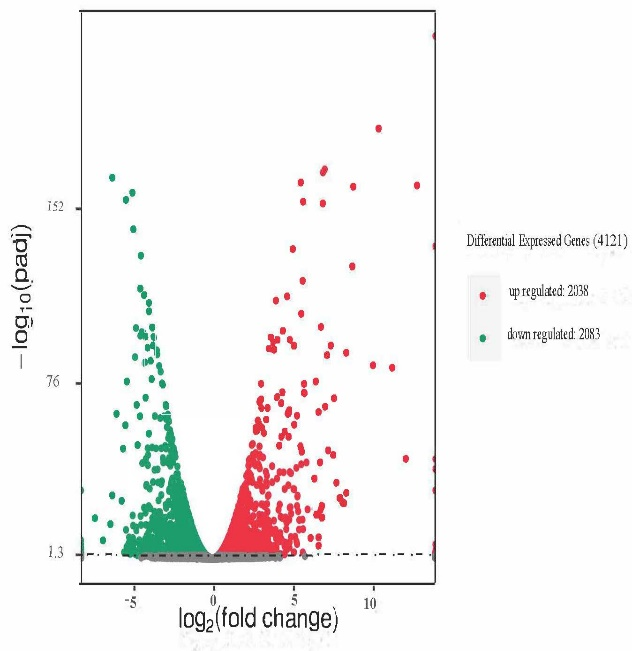


**Figure S3** Gene expression profiles in the mycelium cultured with ammonium sulphate as a nitrogen source in stationary and shaker cultures. (A) The numbers of up- and down-regulated genes. (B) Volcano-plot showing relationship between log_2_ fold-change (log_2_ FC) and FDR.

**
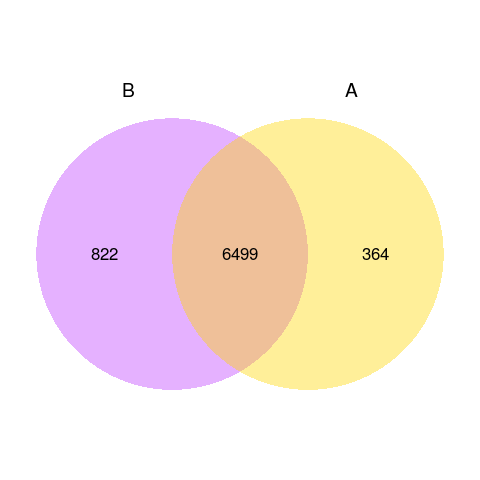
**

**Figure S4** Venn diagram of the number of co-expressed genes based on the mycelium cultured with ammonium sulphate as a nitrogen source in stationary and shaker cultures.
