# Supplementary figures and images for: Exploiting the roles of nitrogen sources for HEA increment in Cordyceps cicadae
Source: Front Microbiol. 2024 May 13;15:1384027. doi: 10.3389/fmicb.2024.1384027 (PMC11129637; doi:10.3389/fmicb.2024.1384027)

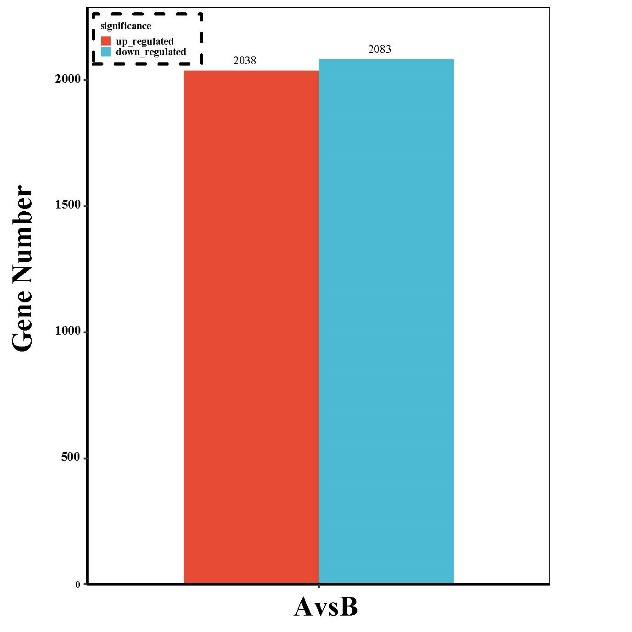

Supplement: Supplementary file 4 [file Image_3.TIF]

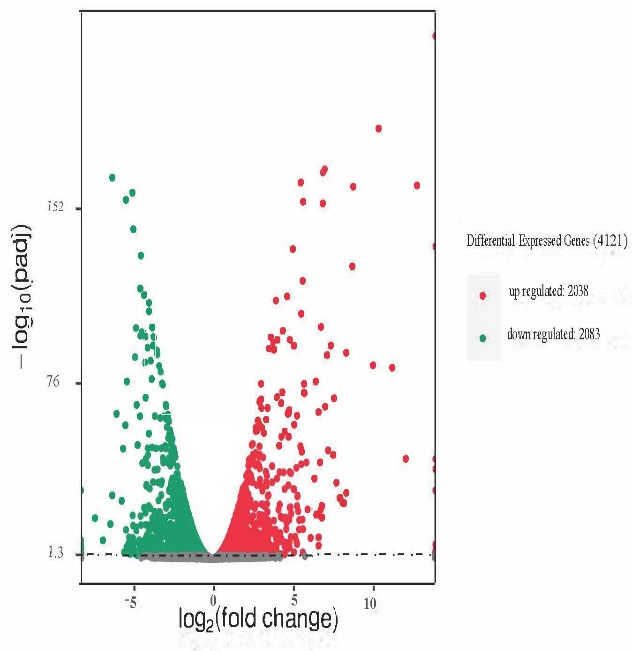

Supplement: Supplementary file 5 [file Image_4.TIF]

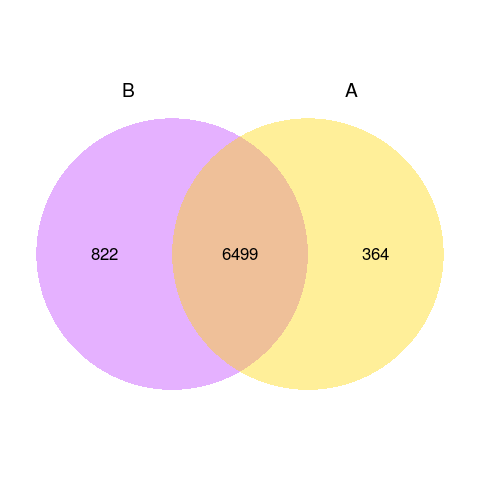

Supplement: Supplementary file 6 [file Image_5.TIF]
